# A carrier-assisted ChIP-seq method for estrogen receptor-chromatin interactions from breast cancer core needle biopsy samples

**DOI:** 10.1186/1471-2164-14-232

**Published:** 2013-04-08

**Authors:** Wilbert Zwart, Rutger Koornstra, Jelle Wesseling, Emiel Rutgers, Sabine Linn, Jason S Carroll

**Affiliations:** 1 Department of Molecular Pathology, The Netherlands Cancer Institute, Amsterdam, the Netherlands; 2 Department of Pathology, The Netherlands Cancer Institute, Amsterdam, the Netherlands; 3 Department of Surgical Oncology, The Netherlands Cancer Institute, Amsterdam, the Netherlands; 4 Department of Medical Oncology, The Netherlands Cancer Institute, Amsterdam, the Netherlands; 5 Cancer Research UK, Cambridge Research Institute, Li Ka Shing Centre, Robinson Way, Cambridge CB2 0RE, UK; 6 Department of Oncology, University of Cambridge, Cambridge CB2 OXY, UK

## Abstract

**Background:**

The Estrogen Receptor alpha (ERα) is the key transcriptional regulator in luminal breast cancer and is therefore the main target for adjuvant treatment of this subtype. Luminal gene signatures are dictated by the transcriptional capacities of ERα, which are a direct consequence of the receptors binding preference at specific sites on the chromatin. The identification of ERα binding signatures on a genome-wide level has greatly enhanced our understanding of Estrogen Receptor biology in cell lines and tumours, but the technique has its limitations with respect to its applicability in limited amounts of tumour tissue.

**Results:**

Here, we present a refinement of the ChIP-seq procedures to enable transcription factor mapping on limited amounts of tissue culture cells as well as from a limited amount of tumor tissue derived from core needle biopsies. Our approach uses a carrier that can be removed prior to DNA amplification and sequencing.

**Conclusion:**

We illustrate the applicability of this refined technology by mapping the ERα genome-wide chromatin binding landscape in core needle biopsy material from primary breast tumours. With this, our refined technology permits for a high-resolution transcription factor mapping even from clinical samples.

## Background

Breast cancer is the most common diagnosed malignancy in women, with over 1.4 million new cases worldwide annually [1]. 75% of all breast cancers are from the luminal subtype [2] for which tumour growth is thought to be dependent on the activity of the Estrogen Receptor alpha (ERα). This growth-dependency renders this pathway the main target for adjuvant endocrine treatment in luminal breast cancer.

The physiological behaviour of the ERα involves binding of the receptor to its natural ligand estradiol, after which the receptor associates to the chromatin, recruits its coregulators and alters the transcriptional activity of responsive genes, leading to increased cell proliferation and tumour growth. In endocrine treatment of breast cancer, the activation of the receptor can be inhibited through multiple ways, each of which resulting in an inhibition of ERα-driven cell proliferation. Even though these endocrine intervention therapies greatly increase the disease-free survival and overall survival of breast cancer patients [3], resistance to treatment is commonly observed. This resistance can be mediated through a multitude of different mechanisms, including differential expression of kinases [4,5], coregulators [3,6] and transmembrane receptors [7,8].

Importantly, it is becoming apparent that mechanisms of resistance may result from intrinsic effects on ERα/chromatin interactions, as shown both in cell lines [9,10] and in tumour samples [11]. Since the ERα/chromatin interactome directly determines the estradiol-mediated effects on gene expression [12], distinct binding patterns may be a key-defining factor for deviating gene expression patterns as well [13]. A major hurdle to assay such transcription factor/chromatin interactions, is the frequently very limited starting material due to the minimal amount of tissue that is obtained during diagnostic work-up.

Recently approaches have been described to map histone modifications in a limited amount of starting material [14], but no such protocol has been described for transcription factors. Similarly, new methods for amplification of limited material have been described [15], but these assume that sufficient DNA was recovered from the ChIP to enable amplification in the first place. One of the major technical limitations has been enriching sufficient DNA (via ChIP) from limited starting material. This problem has been addressed using ‘carrier chromatin’ [16], a strategy that is not amenable to global sequencing approaches. Here, we describe a procedure to enhance transcription factor ChIP-seq by incorporating a modified carrier method to enable ChIP-seq from limited starting material. We apply this method to map the ERα/chromatin interactome in as few as 10,000 tissue culture cells and apply this technology to map the ERα chromatin binding landscape in core needle biopsy samples from breast cancer patients.

## Results and discussion

### Carrier selection for small cell number ChIPs

**Figure 1:**
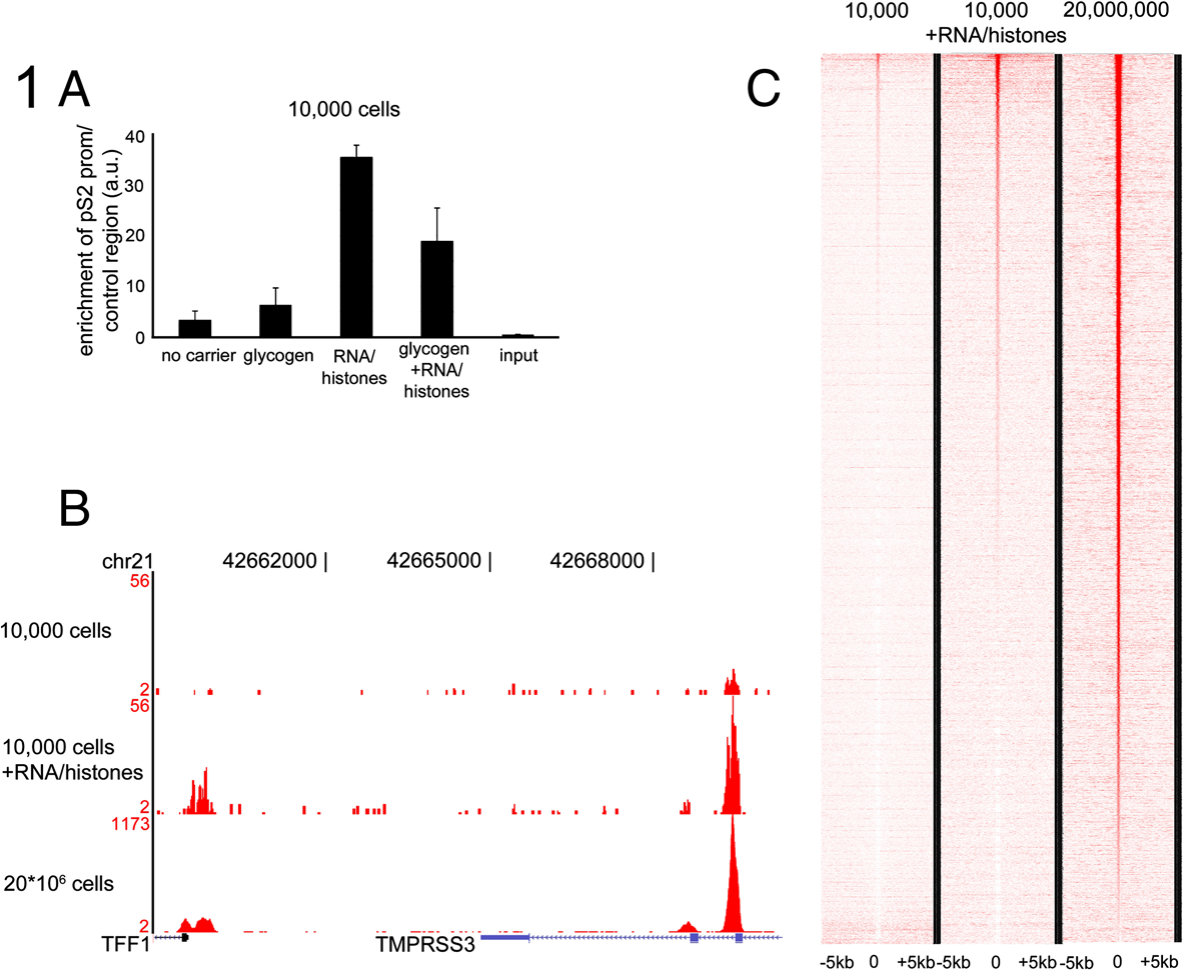
**Carrier-optimized ChIP-seq in MCF7 cells. A**. ChIP-qPCR on pS2/TFF1 promoter for ERα with differential carrier conditions (none, glycogen, mRNA/histones, glycogen/mRNA/histones). Data were normalized over negative control region and input. Bars show standard deviation from triplicate measurements. **B**. Genome browser snapshot of ERα ChIP-seq, depicting the pS2/TFF1 promoter and enhancer region for 10,000 cells without (top) or with mRNA/histones carrier (middle). Data was compared to a saturated ERα ChIP-seq from 20*10^6^ cells (bottom). Genomic coordinates and tag count are indicated. **C**. Heatmap visualization of ERα ChIP-seq data, depicting all binding events centred on the peak region within a 5 kb window around the peak. All peaks for the 20*10^6^ cells ChIP-seq condition were ranked on intensity, and the data from both 10,000 cells-conditions were plotted in identical order.

To optimize the Chromatin Immunoprecipitation (ChIP) procedure from a limited amount of cells, we utilized the well-characterized (ERα-positive and Progesterone Receptor-positive) breast cancer cell line MCF7. MCF7 cells have been widely applied for ChIP-seq applications, and multiple datasets are publicly available to be used as a reference. Proliferating MCF7 cells from a sub-confluent 10 cm dish were harvested, after which the total amount of cells were counted. The lysate from 10,000 cells was used for ChIP. Initially a focused ERα ChIP-real time PCR approach was taken, using the well-studied pS2 promoter region. ERα ChIP was performed using standard methods [17], but in the absence of carrier, no enrichment from the ChIP sample was observed (Figure 1A). In addition, two independent carriers were tested: glycogen and mRNA/histones. The rationale was to test carriers that were either inert (glycogen; not having any effect on the chromatin nor sequencing procedure) or had a composition that could be removed from the sample (mRNA and histones; to be removed by RNAse A and Proteinase K). The carriers were added to the lysate mixture before antibody-conjugate addition. Glycogen proved to have a small, but not significant increase in ChIP efficiency, when using the pS2 promoter region. The addition of recombinant histones and random human mRNA led to a significant increase of ERα signal at the pS2 promoter (Figure 1A). This enhanced enrichment was achieved both by an increased ChIP efficiency for specific signal as well as a decrease of the nonspecific background binding (Additional file 1: Figure S1). Combining the two carrier procedures resulted in a decreased signal as compared to only adding mRNA/histones.

Even though the increased enrichment at the pS2 promoter was indicative of increased ChIP efficiency, this cannot be generalized towards all ERα/chromatin interactions. Therefore, the ERα ChIP-samples were further processed and prepared for sequencing. Only the two extreme conditions (no carrier versus mRNA/Histones carrier) were sequenced and compared to a publicly available dataset of proliferating MCF7 cells, performed on ~20 million cells [10]. The carrier mRNA and histone were degraded prior to amplification of enriched ChIP DNA. An example of difference in binding capacity in the presence and absence of carrier mRNA/histones is shown in Figure 1B and all binding events are illustrated as a heatmap in Figure 1C. Importantly, we ranked all ERα binding peaks observed in the sample with ~20 million cells and found that ~60% of these were recapitulated when using the mRNA and histones as carrier from 10,000 cells. This is a substantial improvement compared to ERα ChIP-seq without carrier, where only signal was detected at ~20% of peaks. Heatmap visualisations and quantifications indicated that peak calling on 10,000 cells with RNA/histones carrier provided comparable signal intensity distributions as the saturated ChIP. This in contrast to the 10,000 cells analysis without carriers (Additional file 2: Figure S2) where mainly background was found. Grouping the ChIP distributions on the basis of signal intensities showed that peaks with high-intensity signal under the RNA/histones carrier ChIP conditions, consistently provided high signal distributions for the saturated ChIP, and vice versa (Additional file 3: Figure S3). No subsets of false-positive and false-negative regions were observed (Additional file 3: Figure S3). Interestingly, the dynamic range of signal intensity as detected in the saturated ChIP was dampened under the carrier conditions (Additional file 3: Figure S3A). Cumulatively, these data show that the addition of carrier chromatin facilitates transcription factor ChIP-seq from 10,000 cells, without negatively effecting the amplification or global fidelity of the process.

**Figure 2:**
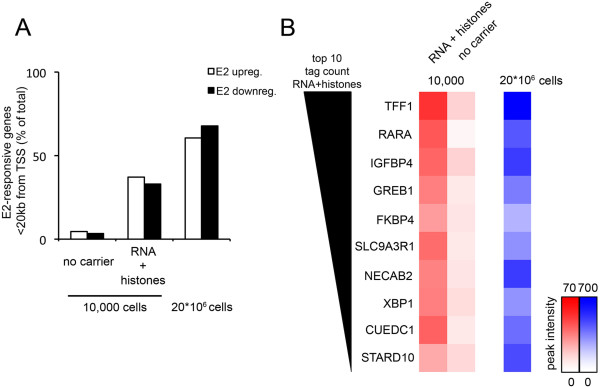
**Proximal Estradiol-responsive genes and carrier ERα ChIP-seq enrichment. A**. Estradiol up- (white) and down-regulated (black) genes were interrogated for proximal ERα binding events under saturated conditions with 20*10^6^ cells (right), as well as ChIP-seq on 10,000 cells without (left) and with mRNA/histones carrier (middle). The percentage of E2-reponsive genes with a proximal ERα binding site is shown. **B**. ERα binding events with mRNA/histones carriers were ranked and proximal E2-upregulated genes were determined for the top 10 highest intensity binding sites. Heatmap indicates tag count.

In order to confirm the biological validity of the ERα binding events mapped using mRNA/histone as carrier for ChIP-seq from 10,000 cells, we integrated the binding information with estrogen (E2)-mediated gene expression profiles. For all E2-regulated genes, we determined whether our altered ChIP protocol enabled the identification of proximal ERα as compared to the saturated ChIP (Figure 2). We used a publically available gene expression microarray dataset, from MCF7 cells treated for 6 hours with E2 [13] and found 1,821 transcripts up-regulated and 1,812 down-regulated by E2. A window of 20 kb around the transcription start-site was applied to determine proximal ERα binding (which has previously been shown to be the optimal window between ERα binding events and promoters of target genes [12]). For the saturated ChIP-seq, approximately 60% of all E2 up- and downregulated genes had a proximal ERα binding event (Figure 2A), which is consistent with our previous report [13]. Using the ERα binding events discovered from 10,000 cells with the mRNA/histones as carrier, we found that 38% of the E2 up-regulated genes had an adjacent ERα binding event (Figure 2A), a figure under-represented because of the fewer ERα binding events in this category. We also ranked the strongest gene-proximal ERα binding events from the mRNA/histone carrier-sample based on tag count, resulting in binding at well-described ERα target genes, including TFF1, RARA, GREB1 and XBP1 (Figure 2B).

### Carrier-ChIP from core needle biopsies of human breast tumours

**Figure 3:**
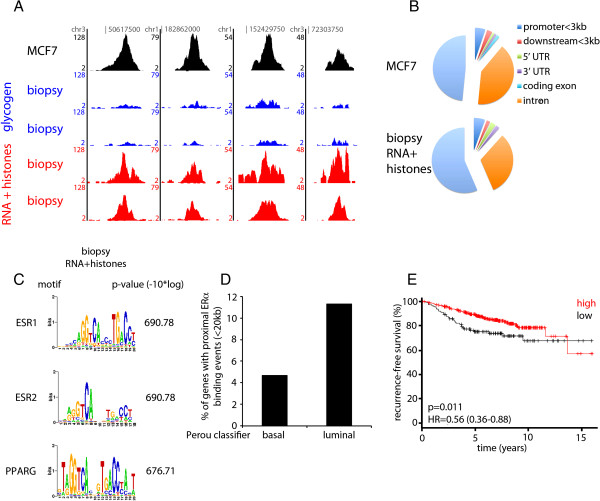
**Carrier ChIP-seq analyses on breast tumour core needle biopsies. A**. Genome browser snapshots of ERα ChIP-seq on MCF7 cells (black) and breast cancer core needle biopsy samples with glycogen (blue) or mRNA/histones (red) as carriers. Tag count is shown on the Y-axis. Genomic locations are indicated. **B**. Genomic distributions of ERα binding events in MCF7 cells (top) and breast tumour biopsy samples with mRNA/histones as carrier (bottom). **C**. Motif analysis of ERα binding events on a core needle biopsy sample, with mRNA/histones as carrier. Indicated are the top 3 enriched motifs and p-values. **D**. Luminal enrichment for ERα carrier ChIP-seq genes. Genes, identified as ‘luminal’ or ‘basal’ signatures by Perou et al. [2] were analysed for proximal ERα binding events. Enrichment of ‘luminal’ over ‘basal’ genes was found. **E**. Genes, identified in **D**., were tested for correlation with recurrence-free survival, using Estrogen Receptor positive samples from a meta-analysis of breast cancer patients [19]. Expression of identified genes correlated with a favourable outcome after endocrine treatment.

Given the findings that mRNA/histone carrier appeared to assist ChIP-seq from limited cell numbers, we applied this approach to clinically relevant samples. We chose to study core needle biopsy samples from breast cancer patients, since they represent clinically available, but limited material that would be appropriate for our approach. These samples were all positive for Estrogen Receptor, as determined by immunohistochemistry (Additional file 4: Table S2). We tested the two carrier conditions independently; 1. glycogen 2. mRNA in combination with recombinant Histone 2B. ERα ChIP-seq was performed in all core needle biopsies. When using glycogen as a carrier, a small level of enrichment was observed from the biopsy samples, as exemplified at multiple binding sites (Figure 3A). Importantly, in the presence of mRNA/histone carriers, the enrichment at both the example regions and globally, was significantly increased. This resulted in 669 ERα peaks in the presence of glycogen and 3,366 peaks in the presence of mRNA/histone carrier for two biopsies with directly comparable clinical parameters (Additional file 4: Table S2). Importantly, in the presence of mRNA/histone carrier, ERα ChIP-seq signal was comparable to the level observed in the cell line (Figure 3A), suggesting that the presence of the carrier enables increased ERα ChIP-seq signal, even when applied to limited samples obtained from core needle biopsies.

ERα binding events are rarely found at promoters (5%) and the majority of binding sites is normally enriched at introns and distal intergenic regions [18]. We analysed the binding site localizations and found that the mRNA/histones assisted ChIP-seq showed comparable genomic distributions compared to cell line data (Figure 3B). Motif analyses were performed for the mRNA/histone assisted ChIP condition (Figure 3C). As expected, ER DNA binding motifs were the most strongly enriched motifs.

Since ERα is the driving transcription factor in luminal breast cancer, we interrogated the genes proximal to the ERα binding events, and determined whether these were previously identified as basal or luminal gene signatures in independent cohorts of breast cancer patients [2] (Figure 3D). For the glycogen carrier condition, none of these hallmark genes were found to have a proximal ERα binding event for any of the tumour samples tested (data not shown). For the mRNA/histone-assisted condition, an enrichment of luminal genes was found over basal-signature genes (Figure 3D). Expression of these signature genes with proximal ERα binding events was found to correlate with a favourable outcome after endocrine treatment in a meta-analysis of breast cancer patients with ERα-positive disease [19] (Figure 3E), indicating that our carrier ChIP-seq protocol enabled us to identify direct ERα target genes with clinical implications from core needle biopsy samples.

## Conclusion

The genome-wide assessment of ERα/chromatin interactions has been performed in tissue culture cell lines [18] and primary breast cancer tissue [11]. Even though ChIP-seq is a very powerful technology to delineate transcription factor-chromatin interactions and to identify specific locations of histone marks on a genome-wide level, the technology does have its intrinsic limitations. The availability of a specific antibody and applicable for immunoprecipitation from formaldehyde-fixed material are major limitations. In addition, there are potential issues related to epitope masking. Another major limitation of ChIP-seq analyses is the need for a certain amount of starting material. Reports have described ChIP-seq analyses on histone marks on limited starting material [14], but these represent relatively stable covalent interactions, as compared to the dynamic and unstable interactions between transcription factors and chromatin. In directed ChIP analyses, the benefit of carrier during the immunoprecipitation procedure has proven to be substantial [16]. In that method, non-relevant chromatin (e.g. drosophila DNA), is added as a carrier during the procedure, in order to enhance the enrichment from the limited but relevant cellular material. While this approach can be applied for qPCR-ChIP, it is not readily applicable for ChIP-seq, which will sequence all DNA, regardless of the species, resulting in substantial dilution of the relevant enriched DNA. How the addition of the carriers exactly enhances the ChIP procedure on a molecular level still needs to be resolved. Most likely, the bulky material of the carrier helps to retain the small amounts of relevant chromatin throughout the isolation procedure. In addition, it might prove an additional benefit of blocking nonspecific antibody/protein interactions, thereby increasing substrate specificity of the antibody in the reaction to reduce background signal.

Here, we introduce a novel mode of carrier-assisted Chromatin Immunoprecipitation that can be applied to transcription factor mapping by ChIP-seq. The enrichment from limited cellular material is enhanced by the addition of mRNA and recombinant histone protein, which function as a carrier. Importantly, both components of the carrier can subsequently be removed and provide an approach that is cost effective, easily applied and applicable in next-generation sequencing reactions.

We applied this approach to map ERα in 10,000 asynchronous MCF7 cells, which results in an improved DNA recovery, when compared to no carrier. Although this recapitulated ~60% of the signal observed when performing ERα ChIP-seq in saturating numbers of cells, these possessed all the expected characteristics of robust ERα binding events. The dynamic range of the ChIP signal intensity was dampened under carrier conditions, possibly implying that weaker ChIP enrichment regions are enhanced in our method. Furthermore, as a proof-of-principle, we applied our carrier-assisted ChIP protocol to limited material derived from core needle biopsy samples, isolated from breast cancer patients. Again, the presence of the mRNA/histone carrier resulted in increased ERα ChIP-seq signal, allowing for discovery of clinically and biologically relevant ERα regulatory domains from these samples. Several approaches have been recently described [15,20] that enhance amplification from limited DNA. Although these are powerful methods, they assume that there is sufficient DNA enriched by ChIP, but currently, methods do not exist that enable the ChIP enrichment from limited material for integration with these novel amplification approaches. We believe that our modified carrier ChIP-method improves the ability for purifying transcription factor-associated DNA from limited material, such as tumour samples. The potential exists for coupling our modified ChIP approach with the novel amplification methods for greater ChIP-seq fidelity and signal from primary material, potentially permitting high-resolution transcription factor mapping even from clinical samples.

## Methods

### Chromatin Immunoprecipitations

ChIP was performed as described before [17], but with a number of modifications. During the Immunoprecipitation, 100 μg/ml glycogen (Invitrogen) or 20 μg/ml recombinant histone 2B (M2505S; New England Biolabs) and 1 μg/ml human mRNA (Invitrogen) was added as carriers for the ChIP. During the entire procedure, non-sticky eppendorf tubes were used (13-698-794; Fisher Scientific). 5 μg antibody used was raised against ERα (SC-543; Santa Cruz), conjugated to 50 μl protein A Dynabeads (Invitrogen). After immunoprecipitation, ten RIPA washes were performed (samples transferred to a fresh eppendorf during the last wash), followed by one TBS wash and reverse crosslinking, as described [17].

### Solexa sequencing

ChIP DNA was amplified as described [17], using 18 PCR amplification cycles. Sequences generated by the Illumina GAIIx genome analyzer (using 36-bp reads) were aligned against NCBI Build 36.3 of the human genome using MAQ (http://maq.sourceforge.net/) with default parameters. Peaks were called using model-based analysis for ChIP-Seq (MACS)[21] run using default parameters. Read counts and duplication rates for all cell line and tumor samples are shown in Additional file 5: Table S1. The number of clonal reads in the carrier sample was higher and may be a result of increased duplication reads of remaining non-ligated adapters.

### Motif analysis, heatmaps and genomic distributions of binding events

ChIP-seq data snapshots were generated using the Integrative Genome Viewer IGV 2.1 (http://www.broadinstitute.org/igv/). Motif analyses were performed through the Cistrome (cistrome.org), applying the SeqPos motif tool [22]. The genomic distributions of binding sites were analysed using the *cis*-regulatory element annotation system (CEAS) [23]. The genes closest to the binding site on both strands were analysed. If the binding region is within a gene, CEAS software indicates whether it is in a 5^′^UTR, a 3^′^UTR, a coding exon, or an intron. Promoter is defined as 3 kb upstream from RefSeq 5^′^ start. If a binding site is >3 kb away from the RefSeq transcription start site, it is considered distal intergenic.

### Tumour tissue handling

Tissue for ChIP-seq analyses was from four newly diagnosed breast cancer patients who were treated at the Netherlands Cancer Institute. Three specimens were collected under a prospective pre-operative endocrine therapy study approved by the institutional ethics committee. These patients provided written informed consent. Also one sample, taken from anonymous left over material, which would be discarded otherwise, was used. Since we are using anonymous, coded leftover material which is not traced back to the patients and does therefore not interfere with care and/ or prognosis, no ethical approval is required according to Dutch legislation [the Medical Research Involving Human Subjects Act; http://www.ccmo-online.nl/main.asp?pid=10&sid=30&ssid=51) and our institutional medical ethical review board. This conforms as described [24]. Three biopsies (14-gauge, 15 μm thick) were taken from each tumour, two of which where frozen with liquid nitrogen and one was formalin-fixed for direct clinical assessment. Tumour cell percentage was determined using Hematoxylin and Eosin (H&E), and tissue was only used for ChIP-seq analysis when tumour cell percentage was higher then 50%. All samples stained positive for Estrogen Receptor. Further information on the tumor samples and patient characteristics can be found in Additional file 4: Table S2.

### Gene expression analyses and survival data

Genes with an ERα binding site within 20 kb from the transcription start site were interrogated. Estradiol-responsive genes were used from a publically available database [13]. Luminal and basal genes were previously identified [2]. For survival analyses, a publically available meta-analysis was used. ERα positive-tumours were selected from breast cancer patients who received adjuvant endocrine treatment [19].

### Data deposition

All genomic data is deposited at ArrayExpress under the accession number E-MTAB-1534.

## Competing interests

The authors declare no competing financial interests.

## Authors’ contributions

WZ carried out the experiments and analyzed the data. RK provided the tissue samples and participated in the design of the study. SL participated in the design of the study, coordinated the clinical aspects of the study and helped to draft the manuscript. ER provided the tissue samples and helped in design the clinical aspects of the study. JW performed the pathological assessment of the tissue samples and helped drafting the manuscript. JC and WZ conceived, designed and coordinated the study and drafted the manuscript. All authors read and approved the final manuscript.

## Supplementary Material

Additional file 1: Figure S1 Carrier ChIP QPCR enrichment for the pS2 promoter and negative control region. ChIP enrichment was calculated as percentage over input, both for the pS2-positive control (left panel) and for the negative control region (right panel). The RNA/histones carrier increases signal as the pS2 positive control region, while it diminishes signal at the negative control site. Click here for file

Additional file 2: Figure S2 Heatmap visualizations and signal quantifications for the three ChIP conditions. MACS peak caller was applied for sequencing data from samples without carrier (**A**), with the RNA/histones carriers (**B**) and the saturated ChIP (**C**). For each peak calling dataset, the corresponding genomic locations were tested for all three sequencing runs, as visualized in heatmaps (arrow head indicated centre of the peak, scale = 5 kb) and quantified in the 2D graphs. Click here for file

Additional file 3: Figure S3. Peak subgroups and correlations between carriers/saturated ChIP samples. MACS peak caller was applied for sequencing data from samples with the RNA/histones carriers (**A**) and the saturated ChIP (**B**). Peaks were subgrouped in ‘high’ (I), ‘medium’ (II) and ‘low’ (III), and raw signal intensity for the RNA/histones carriers and the saturated ChIP sequencing runs was analyzed. For each of the subsets, the corresponding genomic locations were visualized in heatmaps (arrow head indicated centre of the peak, scale = 5 kb) and quantified in the 2D graphs. Click here for file

Additional file 4: Table S2 Tumor characteristics, hormonal status and menopausal state. Click here for file

Additional file 5: Table S1 Sequencing reads and duplication rate. Click here for file
