# Supplementary figures and images for: A carrier-assisted ChIP-seq method for estrogen receptor-chromatin interactions from breast cancer core needle biopsy samples
Source: BMC Genomics. 2013 Apr 8;14:232. doi: 10.1186/1471-2164-14-232 (PMC3637562; doi:10.1186/1471-2164-14-232)

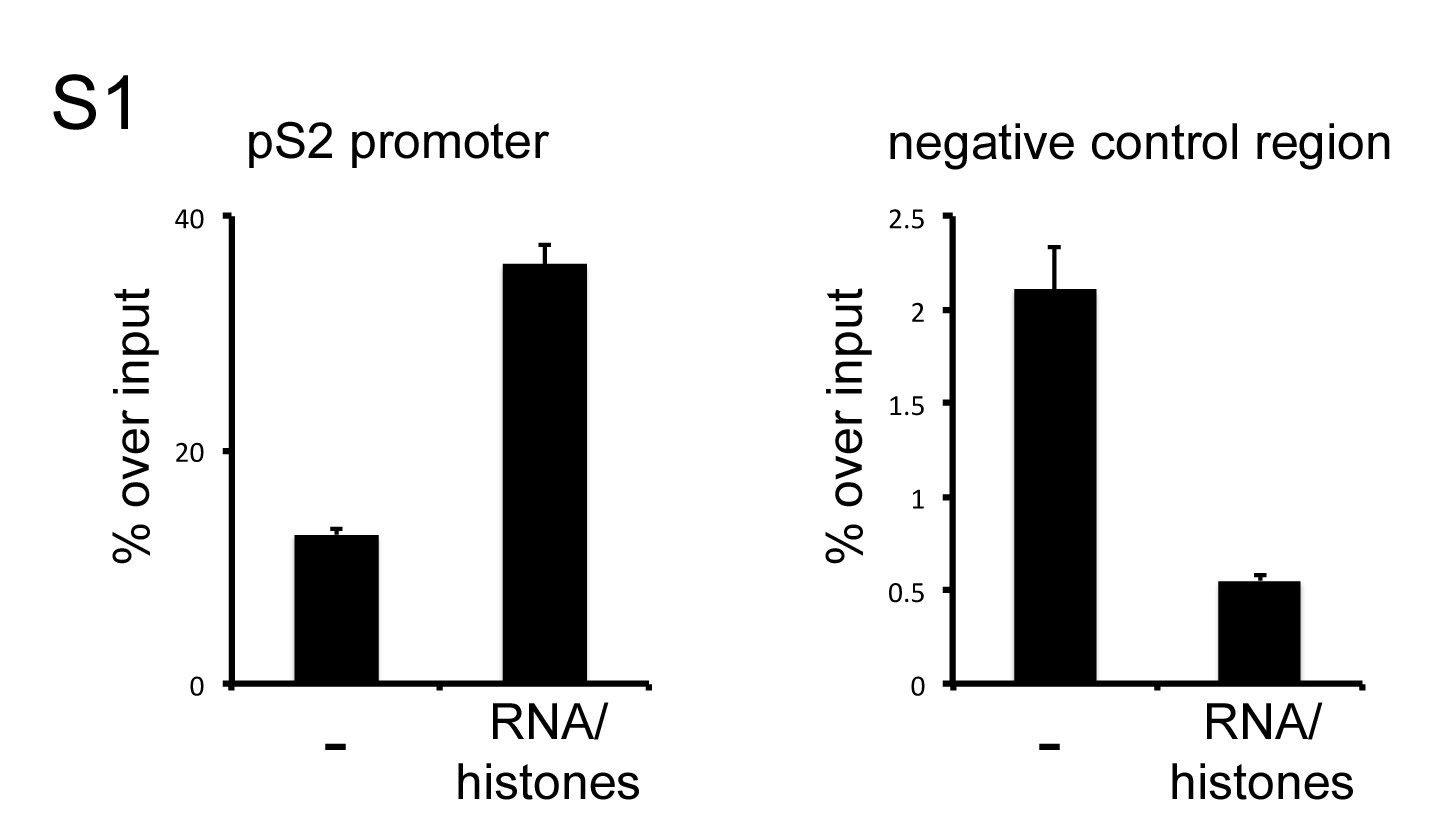

Supplement: Additional file 1: Figure S1 — Carrier ChIP QPCR enrichment for the pS2 promoter and negative control region. ChIP enrichment was calculated as percentage over input, both for the pS2-positive control (left panel) and for the negative control region (right panel). The RNA/histones carrier increases signal as the pS2 positive control region, while it diminishes signal at the negative control site. [file 1471-2164-14-232-S1.jpeg]

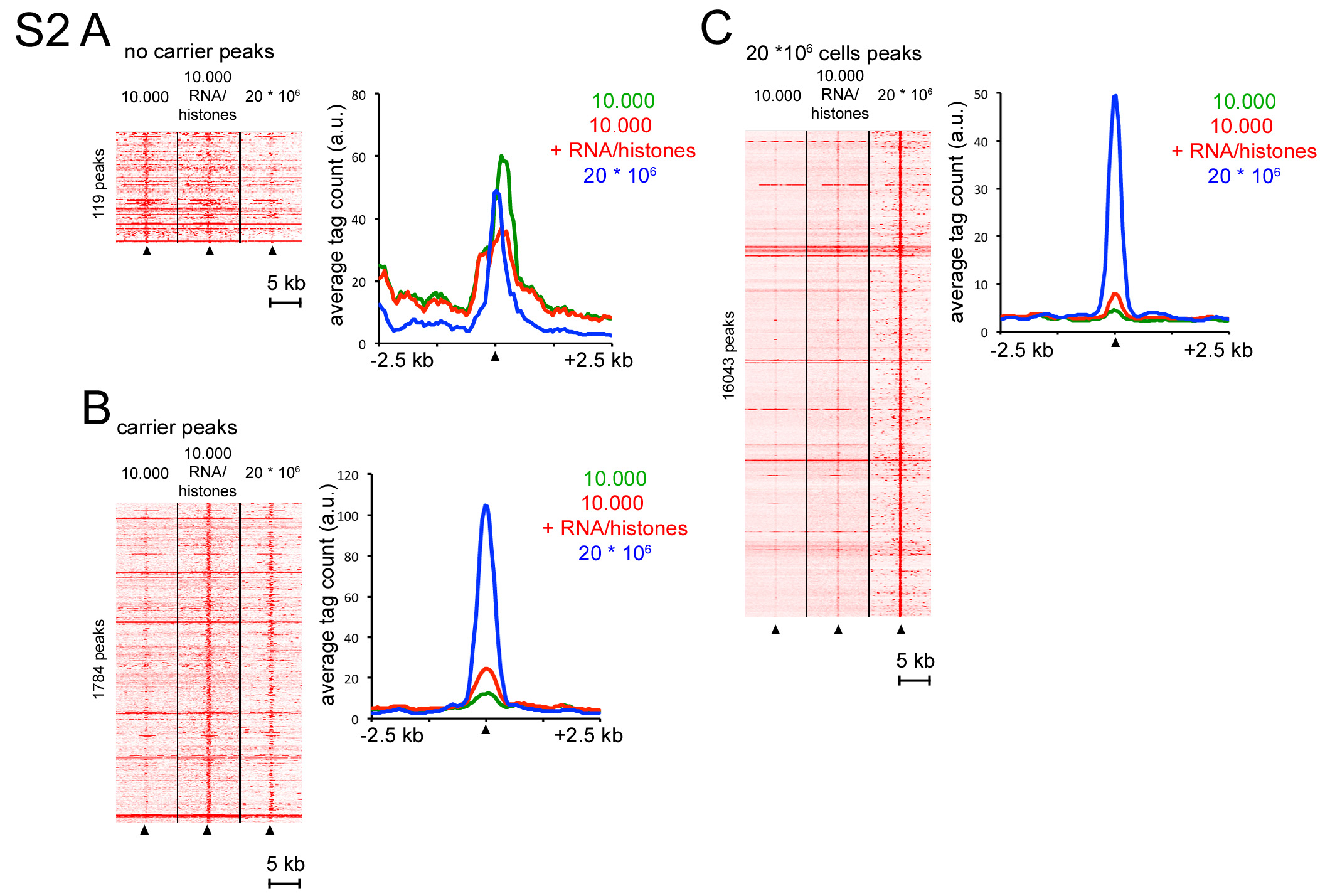

Supplement: Additional file 2: Figure S2 — Heatmap visualizations and signal quantifications for the three ChIP conditions. MACS peak caller was applied for sequencing data from samples without carrier (A), with the RNA/histones carriers (B) and the saturated ChIP (C). For each peak calling dataset, the corresponding genomic locations were tested for all three sequencing runs, as visualized in heatmaps (arrow head indicated centre of the peak, scale = 5 kb) and quantified in the 2D graphs. [file 1471-2164-14-232-S2.jpeg]

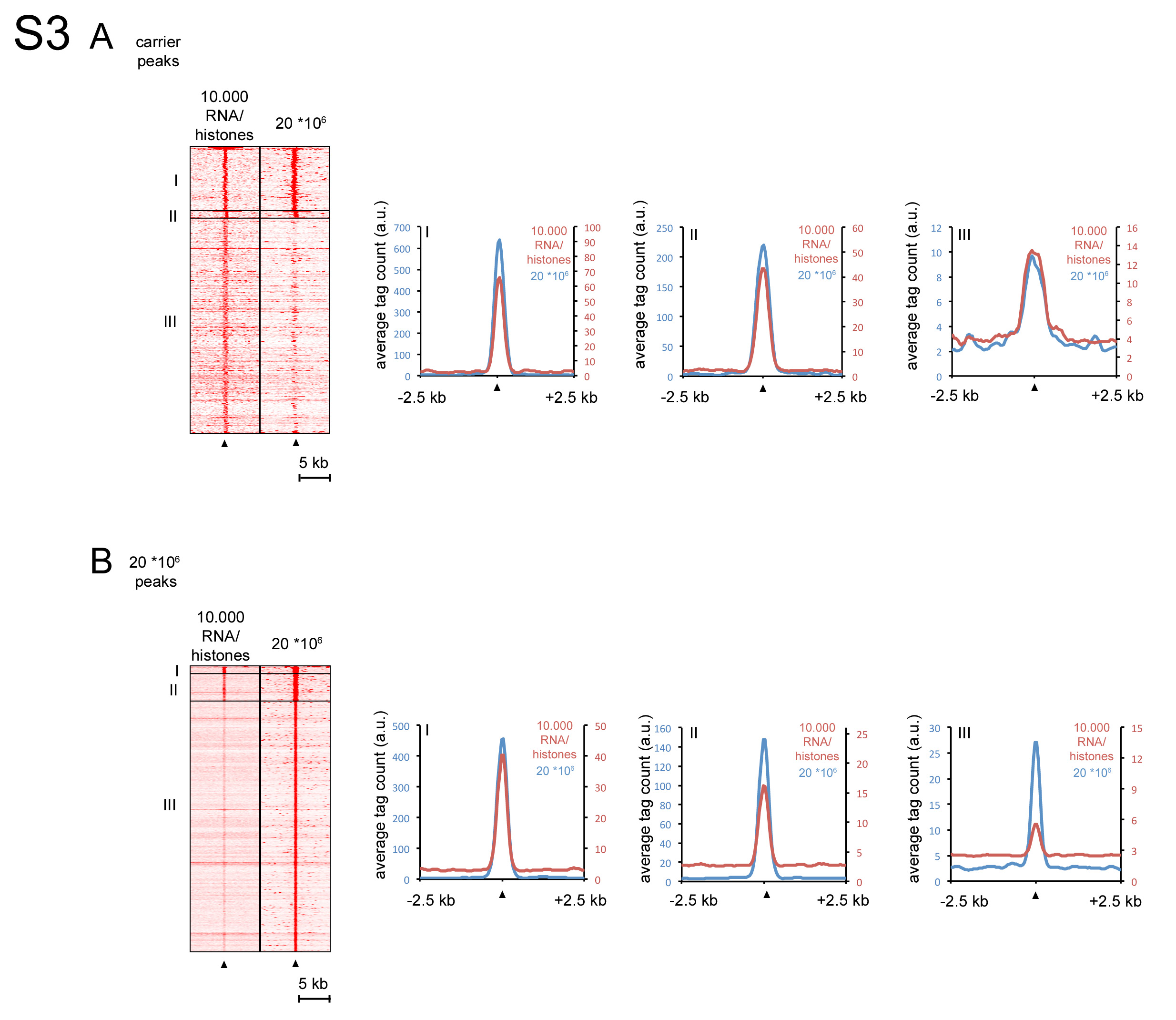

Supplement: Additional file 3: Figure S3. — Peak subgroups and correlations between carriers/saturated ChIP samples. MACS peak caller was applied for sequencing data from samples with the RNA/histones carriers (A) and the saturated ChIP (B). Peaks were subgrouped in ‘high’ (I), ‘medium’ (II) and ‘low’ (III), and raw signal intensity for the RNA/histones carriers and the saturated ChIP sequencing runs was analyzed. For each of the subsets, the corresponding genomic locations were visualized in heatmaps (arrow head indicated centre of the peak, scale = 5 kb) and quantified in the 2D graphs. [file 1471-2164-14-232-S3.jpeg]
