# Supplementary material for: A carrier-assisted ChIP-seq method for estrogen receptor-chromatin interactions from breast cancer core needle biopsy samples
Source: BMC Genomics. 2013 Apr 8;14:232. doi: 10.1186/1471-2164-14-232 (PMC3637562; doi:10.1186/1471-2164-14-232)
Supplement: Additional file 4: Table S2 — Tumor characteristics, hormonal status and menopausal state. [file 1471-2164-14-232-S4.docx]

| Tumor sample |  | ER (%) | PR (%) | menopausal state | |
| --- | --- | --- | --- | --- | --- |
|  |  |  |  |  |  |
| glycogen carrier #1 | | 100 | 80 | postmenopausal | |
| glycogen carrier #2 | | 100 | 0 | postmenopausal | |
|  |  |  |  |  |  |
| RNA/histones #1 | | 100 | 80 | premenopausal | |
| RNA/histone #2 | | 100 | 90 | postmenopausal | |

Supplemental table S2: tumor characteristics
