# Supplementary material for: A carrier-assisted ChIP-seq method for estrogen receptor-chromatin interactions from breast cancer core needle biopsy samples
Source: BMC Genomics. 2013 Apr 8;14:232. doi: 10.1186/1471-2164-14-232 (PMC3637562; doi:10.1186/1471-2164-14-232)
Supplement: Additional file 5: Table S1 — Sequencing reads and duplication rate. [file 1471-2164-14-232-S5.docx]

|  |  |  | Unique |  | Duplicates |  |
| --- | --- | --- | --- | --- | --- | --- |
| sample |  | **Reads** | n | **%** | n | **%** |
| 10.000 cells |  | 20,761,074 | 17,195,583 | 82.8% | 3,565,491 | 17.2% |
| 10.000 cells + RNA/histones | | 23,967,040 | 11,561,574 | 48.2% | 12,405,466 | 51.8% |
| 20.000.000 cells | | 13,633,868 | 12,543,908 | 92.0% | 1,089,960 | 8.0% |
|  |  |  |  |  |  |  |
| tumor #1 glycogen | | 31,904,245 | 21,721,085 | 68.1% | 10,183,160 | 31.9% |
| tumor #2 glycogen | | 28,176,137 | 22,932,202 | 81.4% | 5,243,935 | 18.6% |
| tumor #1 RNA/histones | | 48,326,372 | 15,244,205 | 31.5% | 33,082,167 | 68.5% |
| tumor #2 RNA/histones | | 27,539,302 | 23,549,258 | 85.5% | 3,990,044 | 14.5% |

Supplemental table S1: sequencing reads and duplication rate
